# Vitamin D supplementation does not prevent the recurrence of Graves’ disease

**DOI:** 10.1038/s41598-019-55107-9

**Published:** 2020-01-08

**Authors:** Yoon Young Cho, Yun Jae Chung

**Affiliations:** 1 Division of Endocrinology and Metabolism, Department of Internal Medicine, Gyeongsang National University Hospital, Jinju, Korea; 2 Institute of Health Sciences, Gyeongsang National University School of Medicine, Jinju, Korea; 3 Sungkyunkwan University, Graduate School of Medicine, Seoul, Korea; 4 Division of Endocrinology and Metabolism, Department of Internal Medicine, Chung-Ang University Hospital, Seoul, Korea; 5 Chung-Ang University, College of Medicine, Seoul, Korea

**Keywords:** Thyroid diseases, Outcomes research

## Abstract

Recent literature has reported a higher prevalence of vitamin D deficiency among people with Graves’ disease. No study has examined the effect of vitamin D supplementation on the clinical outcomes of Graves’ disease. We aimed to evaluate whether daily vitamin D supplementation reduces Graves’ disease recurrence. We enrolled 210 subjects with Graves’ disease and vitamin D deficiency and followed them for at least one year after anti-thyroid drug (ATD) discontinuation. Among 210 individuals, 60 (29%) were amenable to taking vitamin D supplements, resulting in sufficient vitamin D levels (from 10.6 to 25.7 ng/mL), whereas the mean vitamin D level was 11.6 ng/mL in the 150 patients who did not take vitamin D supplements. The recurrence rate was similar in both groups (38% vs. 49%, P = 0.086). However, recurrence occurred earlier in the latter group (7 months vs. 5 months, P = 0.016). In the multivariate analysis, vitamin D levels and TSH-binding inhibitory immunoglobulin (TBII) titers at ATD discontinuation remained significant factors for recurrence. Vitamin D levels and TBII titers at ATD discontinuation exhibited a weak negative correlation (R = −0.143, P = 0.041). Vitamin D supplementation might have a protective effect against Graves’ disease recurrence with a borderline significant recurrence rate reduction.

## Introduction

Autoimmune thyroid diseases (AITDs), including Graves’ disease and Hashimoto’s thyroiditis, are the most common organ-specific autoimmune disorders^1^. AITDs are caused by various environmental triggers, such as iodine, drugs, radiation, and infection in genetically predisposed individuals^2^. AITDs are characterized by T-cell-mediated autoimmune diseases, and Graves’ disease is primarily related to hyperactive humoural responses that lead to the production of stimulatory autoantibodies for the thyroid stimulating hormone (TSH) receptor^1^.

Vitamin D regulates bone metabolism and the homeostasis of calcium and phosphorus. The active form of vitamin D binds to the nuclear vitamin D receptor (VDR) and controls the expression of over 200 genes responsible for the regulation of cell proliferation, differentiation, and apoptosis in most tissues and cells, including immune cells^3^. Non-skeletal actions of vitamin D have been studied over the past few decades, and evidence suggests that there is a relationship between vitamin D deficiency and various diseases, such as autoimmune diseases^4^, cardiovascular disease^5,6^, and cancer^5,7^.

Recent studies have reported that low vitamin D levels are prevalent in patients with Graves’ disease. Kivity *et al*. observed a higher prevalence of vitamin D deficiency (defined as 25-hydroxyvitamin(OH)D < 10 ng/mL) in 22 Graves’ disease patients compared with 98 healthy controls (64% vs. 30%, P < 0.01)^8^. Yasuda *et al*. reported reduced levels of vitamin D in 26 Graves’ disease patients compared to the levels in 46 controls (25(OH)D_3_ levels of 14.4 ng/mL vs. 17.1 ng/mL, P < 0.05)^9^. Two meta-analyses implicated vitamin D deficiency as a risk factor for Graves’ disease^4,10^, although the criteria used to define vitamin D deficiency were variable in the studies included in these meta-analyses (25(OH)D < 10–20 ng/mL).

However, studies regarding the role of vitamin D in the consequences of Graves’ disease, such as thyroid function or recurrence, are limited. Yasuda *et al*. found low vitamin D levels in 36 patients without remission compared to the levels in 18 patients in remission (25(OH)D_3_ levels of 14.5 ng/mL vs. 18.2 ng/mL, P < 0.005)^11^. In a prior study, we reported similar vitamin D concentrations in 95 patients with Graves’ disease recurrence and 48 patients in remission (25(OH)D levels of 10.8 ng/mL vs. 11.8 ng/mL, P = 0.405); however, the risk for Graves’ disease recurrence was higher when using the cut-off of 25(OH)D ≤ 14.23 ng/mL (hazard ratio (HR) 3.016, 95% confidence interval (CI) 1.163–7.819, P = 0.023)^12^. Recently, Planck *et al*. observed no difference in clinical parameters, including thyroid function, TSH-binding inhibitory immunoglobulin (TBII), and recurrence rate, in 292 patients with Graves’ disease in a Swedish cohort^13^.

All studies of vitamin D and Graves’ disease were cross-sectional; thus, they had limitations in regard to assessing the association between vitamin D levels and disease consequences. Notably, no study has examined the use of vitamin D among patients with Graves’ disease. Therefore, we investigated the clinical outcomes of Graves’ disease patients, including recurrence rate, one year after anti-thyroid drug (ATD) cessation according to daily vitamin D supplementation status.

## Results

### Demographics according to vitamin D supplementation status

**Figure 1:**
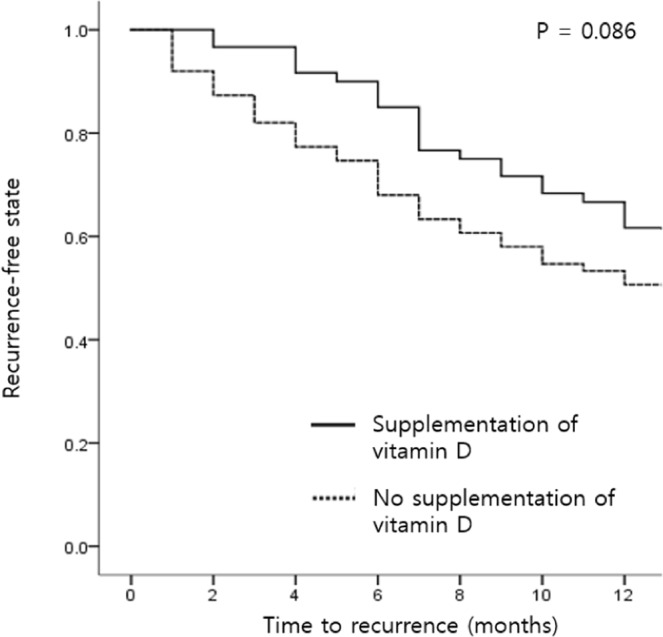
Kaplan-Meier curves for the recurrence of Graves’ disease according to vitamin D supplementation status.

The clinical characteristics of the participants were described according to vitamin D supplementation (Table 1): 60 patients took vitamin D supplements and 150 patients did not take vitamin D supplements. The two groups had similar baseline parameters in terms of age and thyroid function at diagnosis and ATD discontinuation; however, sex (female, 65% vs. 79%, P = 0.015) and TBII levels at the time of ATD discontinuation (0.89 IU/L vs. 1.15 IU/L, P = 0.015) were different between two groups at baseline. In the vitamin D supplementation group, the mean (±SD) vitamin D level increased from 10.6 (±5.4) ng/mL to 25.7 (±3.6) ng/mL. The vitamin D supplementation group took vitamin D supplements for a mean duration of 31 months and at a mean dose of 1383 IU per day; 28 patients (47%) took 1000 IU vitamin D per day, 18 patients (30%) took 1500 IU per day, and 14 patients (23%) took 2000 IU per day. The time to recurrence was delayed in the supplementation group compared to the time to recurrence of the no supplementation group (7 ± 3 months vs. 5 ± 3 months, P = 0.016). However, the difference in recurrence rate within one year was similar with borderline significance (38% vs. 49%, P = 0.086) (Fig. 1).

**Table 1:** Clinical characteristics according to vitamin D supplementation status.

Variables	Supplementation (n = 60)	No supplementation (n = 150)	P
Age, years	41 ± 15	39 ± 12	0.197
Sex, female	39 (65%)	118 (79%)	**0.015**
Thyroid function at diagnosis			
T3, ng/dL	216 (129, 369)	268 (139, 363)	0.537
FT4, ng/dL	2.62 (1.49, 3.89)	2.72 (1.67, 3.69)	0.693
TSH, mU/L	0.01 (0.01, 0.01)	0.01 (0.01, 0.01)	0.990
TBII, IU/L	5.07 (2.94, 14.60)	7.16 (3.94, 11.69)	0.458
Thyroid function at the time of ATD discontinuation			
T3, ng/dL	88 (75, 98)	90 (80, 104)	0.083
FT4, ng/dL	1.17 (1.07, 1.31)	1.14 (1.06, 1.28)	0.494
TSH, mU/L	2.01 (1.11, 3.59)	1.68 (0.93, 2.85)	0.405
TBII, IU/L	0.89 (0.45, 1.32)	1.15 (0.68, 2.22)	**0.015**
Vitamin D supplementation			
Daily dose, IU	1383 ± 405	NA	NA
Duration, months (range)	31 ± 7 (14–36)	NA	NA
Adjusted vitamin D level, ng/mL			<0.001*
Before supplementation	10.6 ± 5.4	11.6 ± 4.9	0.708
After supplementation	25.7 ± 3.6	NA	
Vitamin D measurement, no.	4.0 ± 1.3	1.0 ± 0.2	** < 0.001**
Recurrence within one year after ATD discontinuation	23 (38%)	74 (49%)	0.086
Time to recurrence, months	7 ± 3	5 ± 3	**0.016**

T3, triiodothyronine; FT4, free thyroxine; TSH, thyrotropin; TBII, TSH-binding inhibitory immunoglobulin; ATD, anti-thyroid drug; NA, not available

Variables are presented as number (percentage), mean ± SD, or median (interquartile range).

Significant results (P < 0.05) are indicated in bold.

*P value was calculated based on the supplementation group (vitamin D level after supplementation) vs. the non-supplementation group (vitamin D level before supplementation).

### Vitamin D levels as a protective factor for Graves’ disease recurrence

**Figure 2:**
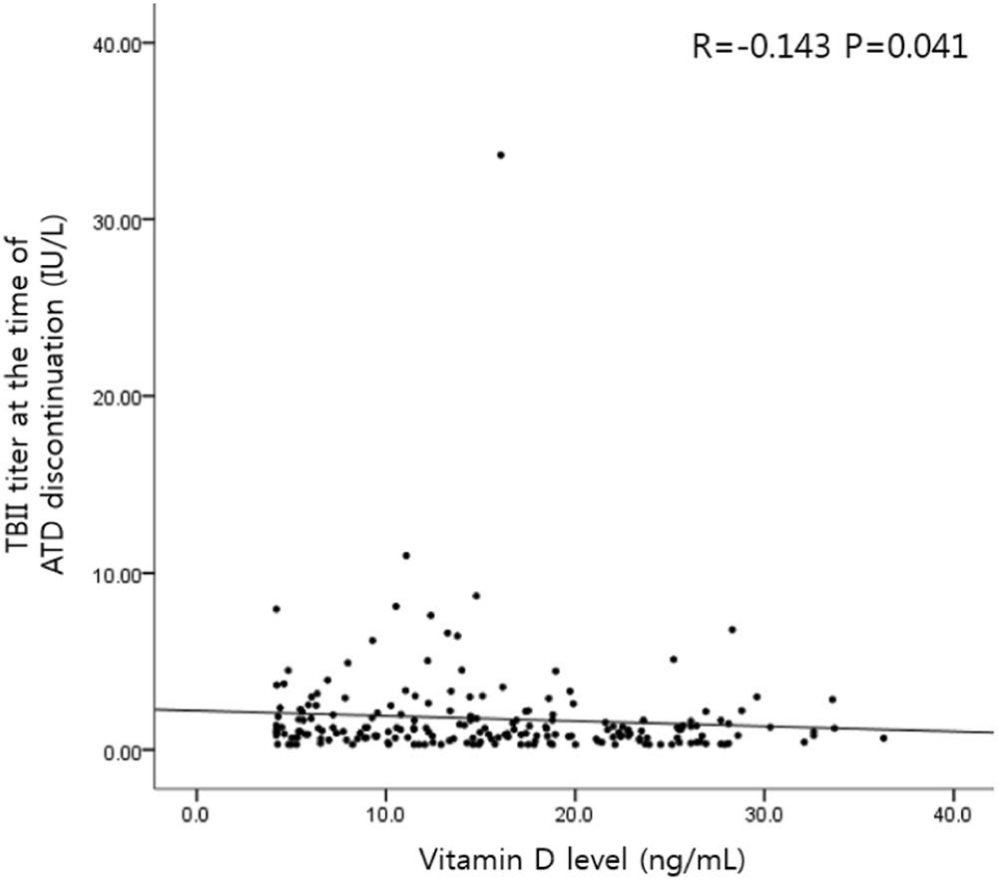
Correlation between vitamin D levels and TBII titers at the time of anti-thyroid drug discontinuation.

We used Cox regression analysis to identify risk factors for the recurrence of Graves’ disease (Table 2). In the univariate analysis, TBII levels at the time of ATD discontinuation (HR 1.07, 95% CI 1.01–1.13, P = 0.018) and vitamin D levels (HR 0.96, 95% CI 0.94–0.99, P = 0.009) were significant risk factors. In the multivariate analysis, the two variables remained significant factors. Vitamin D supplementation was related to the recurrence of Graves’ disease with borderline significance (P = 0.078). Vitamin D levels did not affect thyroid function tests or TBII titers at diagnosis; however, vitamin D levels were related to TBII levels at the time of ATD discontinuation (R = −0.143, P = 0.041) (Table 3 and Fig. 2).

**Table 2:** Cox regression model for the recurrence of Graves’ disease.

Variables	Univariate		Multivariate	
	P	HR (95% CI)	P	HR (95% CI)
Age	0.283		0.354	
Sex	0.937		0.564	
Thyroid function at diagnosis				
T3	0.692		0.533	
FT4	0.710		0.582	
TSH	0.873		0.874	
TBII	0.781		0.704	
Thyroid function at the time of ATD discontinuation				
T3	0.373		0.708	
FT4	0.451		0.441	
TSH	0.691		0.114	
**TBII**	**0.018**	**1.07 (1.01–1.13)**	**0.026**	**1.07 (1.01–1.13)**
Vitamin D supplementation	0.085		0.078	
**Vitamin D level, ng/mL**	**0.009**	**0.96 (0.94–0.99)**	**0.013**	**0.97 (0.94–0.99)**

T3, triiodothyronine; FT4, free thyroxine; TSH, thyrotropin; TBII, TSH-binding inhibitory immunoglobulin; HR, hazard ratio; CI, confidence interval

Significant results (P < 0.05) are indicated in bold.

**Table 3:** Spearman correlation between thyroid function and vitamin D levels.

Variables	P
Thyroid function at diagnosis	
T3	0.658
FT4	0.837
TSH	0.133
TBII	0.724
Thyroid function at the time of ATD discontinuation	
T3	0.154
FT4	0.502
TSH	0.493
**TBII**	0.041*

T3, triiodothyronine; FT4, free thyroxine; TSH, thyrotropin; TBII, TSH-binding inhibitory immunoglobulin; ATD, anti-thyroid drug.

*R (rho): - 0.143.

## Discussion

We demonstrated no beneficial effect of daily supplementation of vitamin D on the recurrence of Graves’ disease within one year after ATD discontinuation. However, the time to recurrence was delayed in patients who reached sufficient levels of vitamin D after vitamin D supplementation. A total of 210 individuals included in our study were vitamin D deficient at study enrolment; among them, 60 subjects were prescribed vitamin D. We considered a level of 25(OH)D ≥ 20 ng/mL as a sufficient vitamin D level after supplementation.

Vitamin D plays an important role in modulating the immune system and the pathogenesis of autoimmune diseases. 25(OH)D (calcidiol) is the inactive but major circulating form of vitamin D and is converted to 1,25(OH)D_2_ (calcitriol), the active form of vitamin D, by 1α-hydroxylase in the kidneys. 1,25(OH)D_2_ binds to the nuclear VDR, which acts on the vitamin D response element (VDRE) of target genes^3^. Recent studies have investigated genetic susceptibilities for the development of Graves’ disease associated with specific variants of single nuclear polymorphisms (SNPs) in VDR genes^13,14^. Aside from the diversity in the genetic background of each individual in terms of vitamin D function, most immune cells are involved in vitamin D action by expressing VDRs, 1α-hydroxylase, and a degrading enzyme^15,16^. 1,25(OH)D_2_ directly enhances the production of Th2 cytokines, whereas it suppresses the production of Th1 cytokines and indirectly shifts the polarization of T cells from a Th1 phenotype to a Th2 phenotype. At the level of antigen-presenting cells (APCs), 1,25(OH)D_2_ inhibits the expression of major histocompatibility complex (MHC) class II antigens and co-stimulatory molecules and prevents the differentiation and maturation of dendritic cells, which leads to decreased antigen presentation and T-cell activation. As one of the pathogenic factors of Graves’ disease, B cells accumulate within thyroid glands and produce thyroid autoantibodies, especially thyroid-stimulating antibodies^17^. 1,25(OH)D_2_ inhibits B-cell proliferation and differentiation into plasma cells and induces B cell apoptosis^18^. These actions of 1,25(OH)D_2_ may suppress thyroid autoantibodies and improve the clinical outcomes of Graves’ disease, which supports our data demonstrating a negative correlation between vitamin D levels and TBII titers at ATD discontinuation, although the correlation power was weak. In brief, active vitamin D, 1,25(OH)D_2_, enhances immune tolerance by suppressing adaptive immunity, which might be beneficial in the context of Graves’ disease. Many studies have shown a higher prevalence of vitamin D deficiency in patients with Graves’ disease than in healthy controls, focusing on the role of vitamin D deficiency in the development of Graves’ disease; however, studies regarding the effects of vitamin D on the clinical course of Graves’ disease are scarce.

The recurrence of Graves’ disease occurred sooner and the recurrence rate tended to be higher in subjects who did not supplement vitamin D than in individuals with sufficient vitamin D levels; however, the difference in the recurrence rate between the two groups was borderline significant. Yasuda *et al*. reported higher vitamin D levels in 18 patients in remission than in 36 patients with active disease (25(OH)D_3_ levels of 18.2 ng/mL vs. 14.5 ng/mL, P < 0.005), but all patients with active disease had positive TBII titers (16.7% vs. 100%, P < 0.0001), suggesting that this may be a determining factor for disease recurrence^11^. In the present study, subjects who supplemented vitamin D achieved vitamin D levels that were twice as high as those of patients who did not supplement vitamin D (25.7 ng/mL vs. 11.6 ng/mL, P < 0.001); however, the TBII titers at the time of ATD discontinuation as well as vitamin D levels were risk factors for the recurrence of Graves’ disease in the multivariate analysis. Planck *et al*. reported no difference in vitamin D levels at baseline between individuals who achieved remission (78 subjects) and those who relapsed (22 subjects) within one year after ATD cessation (vitamin D levels of 22.8 ng/mL vs. 25.3 ng/mL)^13^. In addition, they reported no correlation between vitamin D levels and thyroid hormone levels, including TBII titers. However, previous studies, including that conducted by Planck *et al*., utilized a cross-sectional design, and no prior studies have assessed the role of vitamin D supplementation in the clinical outcomes of Graves’ disease. Furthermore, we derived remission/relapse data from a relatively large number of patients (210 patients vs. 100 in the study by Planck *et al*.^13^).

There might be several plausible explanations for the lack of a definite benefit of vitamin D supplementation on the clinical outcomes of Graves’ disease in our study population. We targeted 25(OH)D levels ≥20 ng/mL, which might be an insufficient level to reduce Graves’ disease recurrence. Vitamin D deficiency is very common in Korea. The recent Korea National Health and Nutrition Examination Surveys (KNHANES) VI-1 and 2 (2013 and 2014) reported that the median 25(OH)D level was 16.0 ng/mL and that only 4% of individuals had a vitamin D level above 30 ng/mL among 4,181 participants^19^. In our previous retrospective study, a lower level of vitamin D (25(OH)D ≤ 14.23 ng/mL) was related to a higher probability of Graves’ disease recurrence^12^, and the current Korean guidelines for osteoporosis recommend 800 IU of daily vitamin D supplementation to maintain levels of vitamin D ≥ 20 ng/mL^20^. The optimal vitamin D concentration for skeletal health is still controversial, although the majority of expert groups adopt 25(OH)D ≥ 20 ng/mL as a sufficient vitamin D level for bone health, based on the trials of vitamin D supplementation and the Institute of Medicine (IOM) systematic review^21–23^; thus, we defined 25(OH)D ≥ 20 ng/mL as sufficient. Although a higher vitamin D level was a preventative factor for disease recurrence in the multivariate analysis, we cautiously assume that vitamin D levels ≥20 ng/mL were not sufficient to prevent the recurrence of Graves’ disease because of borderline significant results of vitamin D supplementation in the multivariate analysis (Table 2) and of recurrence rates between the two groups according to vitamin D supplementation status (Table 1). In addition, the optimal vitamin D level for extra-skeletal health has not been established and may vary according to the organ system. Appropriate serum 25(OH)D levels for disease prevention were different in observational studies, and the ideal level has not been examined for Graves’ disease. For example, the risk of colorectal cancer was lowest in individuals with vitamin D levels >30 ng/mL^24^, and for cardiovascular diseases, the optimal vitamin D level ranged from 20 to 25 ng/mL^6^. Thus, further interventions are needed to identify the optimal vitamin D status for improving clinical outcomes of Graves’ disease. Moreover, vitamin D deficiency might be a less potent factor in the clinical course of Graves’ disease than known risk factors, including TBII titers.

This study has several limitations. First, it was not a randomized controlled trial (RCT); we prescribed daily vitamin D supplementation to those who were amenable to taking it. The timing of vitamin D supplementation initiation also varied; approximately 43% of patients (26/60) started vitamin D supplementation within one year after the initial prescription of ATD, whereas others began thereafter. Therefore, biases such as selection bias may have affected the study results. In addition, regular monitoring of medication compliance was difficult to perform, and the daily dose of vitamin D varied between 1000 and 2000 IU. Thus, we used vitamin D concentrations as an indicator of drug compliance and vitamin D status. Due to the low prevalence of Graves’ disease (2.76 per 1,000 in Korea, 2006–2015)^25^, designing clinical trials for these patients is much more difficult than those with other diseases, such as cancers or cardiovascular disease^5^. We enrolled a considerable number of subjects, and our study is the first to evaluate the clinical impact of vitamin D supplementation in Graves’ disease. Second, we did not compare the vitamin D levels of patients with Graves’ disease with those of healthy controls or consider the genetic aspects of vitamin D function, such as VDR gene expression; however, these topics were beyond the scope of our study. Third, we excluded patients with moderate to severe ophthalmopathy because they accounted for a relatively low percentage of our study population, and interventions for thyroid ophthalmopathy may affect the study results. Thus, our data are not appropriate to evaluate the relationship between thyroid ophthalmopathy and vitamin D status.

This study is the first to examine the effect of vitamin D supplementation on the clinical outcomes of Graves’ disease. Vitamin D levels more than doubled (from 10.6 to 25.7 ng/mL) after daily supplementation of vitamin D, but vitamin D supplementation did not markedly reduce the recurrence of Graves’ disease, although the time to recurrence was somewhat delayed in those with sufficient vitamin D levels. Further interventions are required to examine the effect of higher doses of vitamin D supplementation on Graves’ disease in various populations.

## Methods

### Study population

**Figure 3:**
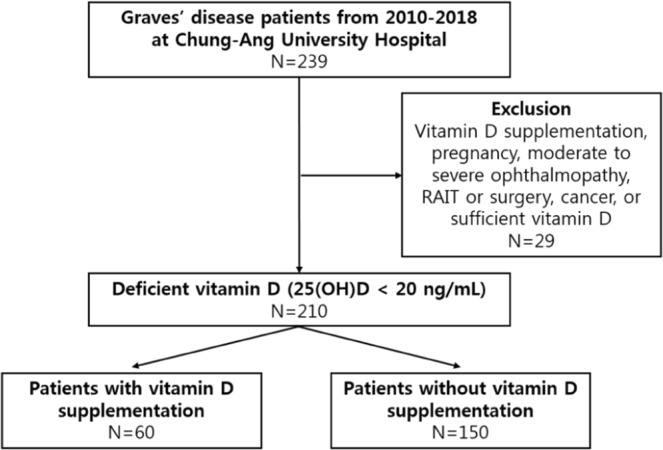
Schematic presentation of the study design.

We enrolled subjects who were treated for Graves’ disease at Chung-Ang University Hospital between November 2010 and July 2018. All patients were taking an ATD and were followed up for at least one year after ATD discontinuation. During ATD treatment, we measured the serum 25(OH)D level. 25(OH)D < 20 ng/mL was defined as vitamin D deficiency, whereas 25(OH)D ≥ 20 ng/mL was defined as a sufficient vitamin D level. To assess the preventative effect of vitamin D supplementation on disease recurrence and maintain homogeneity of the study population, we excluded patients with sufficient vitamin D levels at baseline. We also excluded patients who were already taking vitamin D supplements before study enrolment, those who were pregnant, those who had moderate to severe ophthalmopathy, those with a history of radioactive iodine therapy or surgery for Graves’ disease, and those with a history of cancer. Finally, we included 210 patients with Graves’ disease who were vitamin D deficient at study enrolment (Fig. 3). Approximately half of patients (111/210, 53%) were initially diagnosed with Graves’ disease at our institution, and others (99/210, 47%) were referred from other institutions for a second opinion or for a definite diagnosis of Graves’ disease; among 99 patients, 20 had previously experienced recurrence. Patients amenable to supplementation were prescribed vitamin D_3_ (cholecalciferol) at a dose of 1000–2000 IU per day. As the effect of vitamin D supplementation on the clinical outcomes of Graves’ disease has not been evaluated, we discussed the prescription of vitamin D with each patient and the patient’s preference affected vitamin D supplementation. Among 210 individuals, 60 (29%) were supplied with vitamin D, and the others (150 subjects, 71%) were not. All patients started vitamin D supplementation before ATD cessation. We retrospectively reviewed medical records for age, sex, thyroid function and TBII level at three-month intervals, serum vitamin D concentrations before and after vitamin D supplementation and at the time of ATD discontinuation, and the recurrence of Grave’s disease within one year after ATD discontinuation.

The definition of recurrence was based on thyroid function testing after ATD discontinuation: suppressed thyrotropin (TSH) <0.4 mU/L and elevated free thyroxine (FT4) >1.76 ng/dL and/or triiodothyronine (T3) >181 ng/dL. As recurrence is most likely within the first 6–12 months after ATD withdrawal^26^, remission was defined as the maintenance of normal thyroid function for at least one year after ATD discontinuation according to the definition of the American Thyroid Association^27^. This study was approved by the Institutional Review Board of Chung-Ang University Hospital (no. 1812–017–16229), and all included patients gave their informed consent. All methods were performed in accordance with the relevant guidelines and regulations.

### Laboratory measurements

Serum TSH (reference range, 0.55–4.78 mU/L), FT4 (reference range, 0.89–1.76 ng/dL), and T3 (reference range, 60–181 ng/dL) were measured using a chemiluminescence immunoassay (Siemens Advia Centaur XP, Siemens Healthcare Diagnostics Inc., Tarrytown, NY, USA). The test sensitivities were 0.008 mU/L, 0.1 ng/dL, and 0.1 ng/dL, respectively. The inter-assay coefficients of variation (CV) were <5%, <5%, and <2%, and the intra-assay CVs were <5%, <4%, and <4%, respectively. TBII levels (reference range, 0–1.75 IU/L) were evaluated using an automated electrochemiluminescence immunoassay kit (Elecsys Anti-TSHR, Roche Diagnostics, Mannheim, Germany). The sensitivity was 0.3 IU/L and the inter- and intra-assay CVs were <12% and <8%, respectively.

Levels of 25(OH)D (reference range, 4.2–150 ng/mL) were measured using a chemiluminescence immunoassay (Siemens Advia Centaur XP, Siemens Healthcare Diagnostics Inc.). The sensitivity was 4.2 ng/mL and the inter- and intra-assay CVs were <12% and <8%, respectively. To adjust for seasonal differences in 25(OH)D levels, we included information regarding the season at the time of blood sampling in the models and categorized patients into the following four seasonal groups: June to August (group 1), September to November (group 2), December to February (group 3), and March to May (group 4). The season-adjusted vitamin D levels were calculated by adding residuals from a locally weighted polynomial regression of 25(OH)D of the month of the blood draw to the overall mean value^28^.

### Statistical analysis

Statistical analyses were performed using SPSS Statistics 18 (SPSS Inc., Chicago, IL, USA). Descriptive statistics (mean, SD, median, interquartile range, number, and percentage) were tabulated for clinical characteristics. We performed a chi-square test to compare categorical variables and an independent t-test for parametric variables. We used the Kaplan-Meir test to compare the recurrence-free state of patients with Graves’ disease according to vitamin D supplementation status. We conducted univariate and multivariate analyses using the Cox proportional hazard model to identify risk factors for the recurrence of Graves’ disease. We used the Spearman correlation test to examine the correlation between 25(OH)D levels and thyroid function. We regarded P-values <0.05 as significant and P-values from 0.05–0.1 as borderline significant.

## Data Availability

The datasets generated during the current study are available from the corresponding author on reasonable request.
